# Adverse Outcomes of Anticoagulant Use among Hospitalized Patients with Chronic Kidney Disease: A Comparison of the Rates of Major Bleeding Events between Unfractionated Heparin and Enoxaparin

**DOI:** 10.1371/journal.pone.0106517

**Published:** 2014-09-02

**Authors:** Fatemeh Saheb Sharif-Askari, Syed Azhar Syed Sulaiman, Narjes Saheb Sharif-Askari, Ali Al Sayed Hussain, Mohammad Jaffar Railey

**Affiliations:** 1 School of Pharmacy, Universiti Sains Malaysia, Penang, Malaysia; 2 Pharmacy Department, Dubai Health Authority, Dubai, United Arab Emirates; 3 Nephrology Unit, Dubai Hospital, Dubai, United Arab Emirates; University of Leicester, United Kingdom

## Abstract

**Background:**

Anticoagulation therapy is usually required in patients with chronic kidney disease (CKD) for treatment or prevention of thromboembolic diseases. However, this benefit could easily be offset by the risk of bleeding.

**Objectives:**

To determine the incidence of adverse outcomes of anticoagulants in hospitalized patients with CKD, and to compare the rates of major bleeding events between the unfractionated heparin (UFH) and enoxaparin users.

**Methods:**

One year prospective observational study was conducted in patients with CKD stages 3 to 5 (estimated GFR, 10–59 ml/min/1.73 m^2^) who were admitted to the renal unit of Dubai Hospital. Propensity scores for the use of anticoagulants, estimated for each of the 488 patients, were used to identify a cohort of 117 pairs of patients. Cox regression method was used to estimate association between anticoagulant use and adverse outcomes.

**Results:**

Major bleeding occurred in 1 in 3 patients who received anticoagulation during hospitalization (hazard ratio [HR], 4.61 [95% confidence interval [CI], 2.05–10.35]). Compared with enoxaparin users, patients who received anticoagulation with unfractionated heparin had a lower mean [SD] serum level of platelet counts (139.95 [113]×10^3^/µL vs 205.56 [123] ×10^3^/µL; P<0.001), and had a higher risk of major bleeding (HR, 4.79 [95% CI, 1.85–12.36]). Furthermore, compared with those who did not receive anticoagulants, patients who did had a higher in-hospital mortality (HR, 2.54 [95% CI, 1.03–6.25]); longer length of hospitalization (HR, 1.04 [95% CI, 1.01–1.06]); and higher hospital readmission at 30 days (HR, 1.79 [95% CI, 1.10–2.91]).

**Conclusions:**

Anticoagulation among hospitalized patients with CKD was significantly associated with an increased risk of bleeding and in-hospital mortality. Hence, intensive monitoring and preventive measures such as laboratory monitoring and/or dose adjustment are warranted.

## Introduction

Chronic kidney disease (CKD) affects 10% to 15% of the adult population in United States, Europe, and Asia [1]–[3]. Patients with CKD display a wide range of abnormalities in the homeostatic pathway that may account for their increased risk for both thrombotic events and bleeding [4]. The early stages of CKD are mainly associated with the prothrombotic tendency [4], whereas in its more advanced stages, beside the procoagulant state, platelets can become dysfunctional due to uremic-related toxin exposure leading to an increased bleeding tendency [4], [5].

The increased risk of thromboembolic diseases among CKD patients commonly requires anticoagulation therapy [6]. However, many randomized trials have demonstrated the greater safety and clinical efficacy of low molecular weight heparin (enoxaparin) compared to unfractionated heparin (UFH) in non CKD patients [7]. The ease of use, and the predictable anticoagulant effect of enoxaparin eliminates the need for routine laboratory monitoring [8]. A disadvantage of enoxaparin is its dependence on kidney function for excretion and accumulation of its anticoagulant effect in patients with decreased kidney function [9]; therefore dosage reduction is recommended in patients with severe CKD, defined as creatinine clearance of less than 30 ml/min [10]. To date, it is unknown whether enoxaparin in adjusted therapeutic doses is as safe to prescribe in CKD patients as UFH whose elimination does not depend on the kidney.

In support of these matters, ensuring an accurate enoxaparin dose may have a significant impact on thromboembolic disease outcomes. Therefore, an appropriate therapeutic dose would appear essential in order to maintain a proper balance of efficacy and safety in patients with reduced kidney function. Data from large clinical trials regarding the approval of current enoxaparin dosing have excluded patients with CKD [7], and smaller observational studies [11] have used full therapeutic doses without dose adjustment.

Thus, we conducted a one year prospective study to examine whether the use of anticoagulants (UFH or enoxaparin) for the treatment of thrombotic events in hospitalized patients with CKD was associated with adverse outcomes. Using these data, we first explored the relationship between anticoagulant use and major bleeding events, in-hospital mortality, length of hospital stay, and readmission at 30 days. To limit the potential for confounding by indication, we then examined the association among the subgroup of patients with anticoagulant use and the occurrence of major bleeding events. Finally, we compared the risk of major bleeding in the use of UFH versus adjusted therapeutic doses of enoxaparin.

## Materials and Methods

### Study Design and Participants

This prospective, observational study was conducted at the renal unit of Dubai Hospital, a 625-bed general hospital in Dubai, the United Arab Emirates. Consecutive patients with CKD stages 3 to 5 (estimated glomerular filtration rate [eGFR], 10–59 ml/min/1.73 m^2^) who were admitted to the renal unit, between December 1, 2011, and December 31, 2012 were included. This study was approved by the Medical Research Committee of Dubai Health Authority. The Medical Research Committee did not require a written informed consent from each study participant. However, in case further information was needed, a verbal consent was taken from the respective patient and was documented in the patient data collection form. This consent procedure was approved by the Medical Research Committee.

### Data Collection

For each patient who met the study criteria baseline data was collected on admission and was updated daily by the researcher in charge using a standardized form. Data collected covered demographic characteristics, including age and sex; physical examination results, including blood pressure and weight; comorbid conditions, including diabetes, hypertension, vascular disease, heart failure, and anaemia; laboratory tests, including serum and biochemical parameters; and coadministration of medications taken before admission or during hospital stay that might affect patients bleeding tendencies. The baseline laboratory data was defined as the first test result before the anticoagulant administration. Furthermore, patients risk factors for bleeding such as history of uncontrolled hypertension, cerebrovascular accidents, cancer, falls, or recent surgery were also collected [12].

### Anticoagulant Exposure

In our study, systemic anticoagulants (UFH or enoxaparin) that were administrated for the treatment of deep-vein thrombosis, pulmonary embolism, atrial fibrillation, ischemic stroke, myocardial infarction, unstable coronary artery disease, and acute peripheral arterial occlusion, were included. We defined anticoagulant exposure on the basis of a patient receiving at least 1 course of either UFH or enoxaparin for the treatment of a new thrombotic indication during hospitalization, thus, excluding patients who received only prophylactic doses of either UFH or enoxaparin. We also excluded patients who received concurrent anticoagulation therapy or oral anticoagulants (warfarin sodium) during hospital stay.

In this study, all the anticoagulant drug orders were physician based. Patients baseline laboratory results and body weight were documented before the administration of anticoagulants, and the data was used to calculate the dose according to the British National Formulary [10] and other evidence based guidelines [8]. The doses of enoxaparin were adjusted based on the degree of kidney function. The dosages used were either 1 mg/kg body weight administered subcutaneously every 24 hours or 0.75 mg/kg every 12 hours. The doses of UFH were up to 30,000 units of heparin over 2–3 times per day. The anticoagulation activity of UFH was monitored by measuring the activated partial thromboplastin time (APTT) for all patients daily, and the doses were then adjusted accordingly.

### Adverse Outcomes

The main outcome measures studied were the effect of anticoagulation therapy on, major bleeding events, in-hospital mortality, length of hospital stay, and readmission at 30 days. A major bleeding was defined as overt bleeding resulting in death, transfusion of two or more units of packed blood cells, a fall in haemoglobin level to ≥3 g/dL, the need for corrective surgery intervention, or the occurrence of intracranial, retroperitoneal, or intraocular bleeding [13], [14]. Anticoagulant-related bleeding was defined as bleeding that occurred (1) during UFH or enoxaparin therapy (2) following the discontinuation of UFH or enoxaparin therapy within 24 hours prior to the bleeding events. In-hospital mortality was defined as all cause death occurring during the hospital stay. Readmission was detected by screening for a patient revisit within the specific period.

### Statistical Analysis

Baseline characteristics of patients with and those without anticoagulation treatment were compared by using either a chi-square test for categorical variables and t-test or Mann-Whitney test, depending on skewness of data, for continuously distributed variables.

#### Propensity-Based Matching

Because anticoagulant users may differ in key baseline characteristics from those of non-users, and to allow for an unbiased comparison between these two groups, a propensity score-matching was performed [15], [16]. The propensity scores were estimated using logistic regression with the dependent variable of anticoagulant use and the independent variables selected from baseline characteristics of study cohort. To remove confounding bias, patient variables that were considered as confounders of the association between anticoagulation therapy and major bleeding events were used to create the propensity score [17], [18]. Variables used in the propensity score included: age, sex, estimated GFR, serum albumin, serum platelet counts, Charlson Comorbidity Index score [19], diabetes, hypertension, vascular disease, anaemia, history of gastrointestinal bleeding, history of stroke, and use of aspirin and clopidogrel. Matching was performed using the ‘psmatching’ custom dialogue in conjunction with SPSS version 21 [16]. Study cohorts were matched using nearest neighbour one-to-one matching, without replacement, and a caliber width of 0.2 of the standard deviation. Adequacy of balance for the covariates in the matched samples was assessed using a standardized mean difference between the prematch and postmatch groups, considering differences less than 10% as good balance [20].

#### Outcome Analyses

The outcome analysis was performed comparing these propensity-matched anticoagulant users and non-users. The risk of in-hospital mortality, the occurrence of major bleeding, length of hospital stay, and readmission at 30 days in relation with the anticoagulation therapy, was estimated separately using a Cox proportional hazard regression model that stratified on the matched pairs. The hazard ratio of major bleeding in relation with the use of UFH and enoxaparin was reported graphically using Kaplan-Meier estimates, plotting the log-minus-log survival function over time. The log-rank test was used to investigate the crude association with the use of UFH and enoxaparin and risk of major bleeding.

#### Sensitivity Analyses

The association of anticoagulation therapy with major bleeding events was further explored by stratifying the cohorts by age, sex, history of diabetes, hypertension, vascular disease, estimated GFR, serum level of platelet counts, and treatment with aspirin and clopidogrel. For these (subgroup) analyses, the risk of major bleeding in relation to anticoagulants exposure was estimated separately using proportional Cox regression models that incorporated propensity scores.

All tests were 2 tailed and a P value of less than 0.05 was considered statistically significant.

## Results

**Figure 1:**
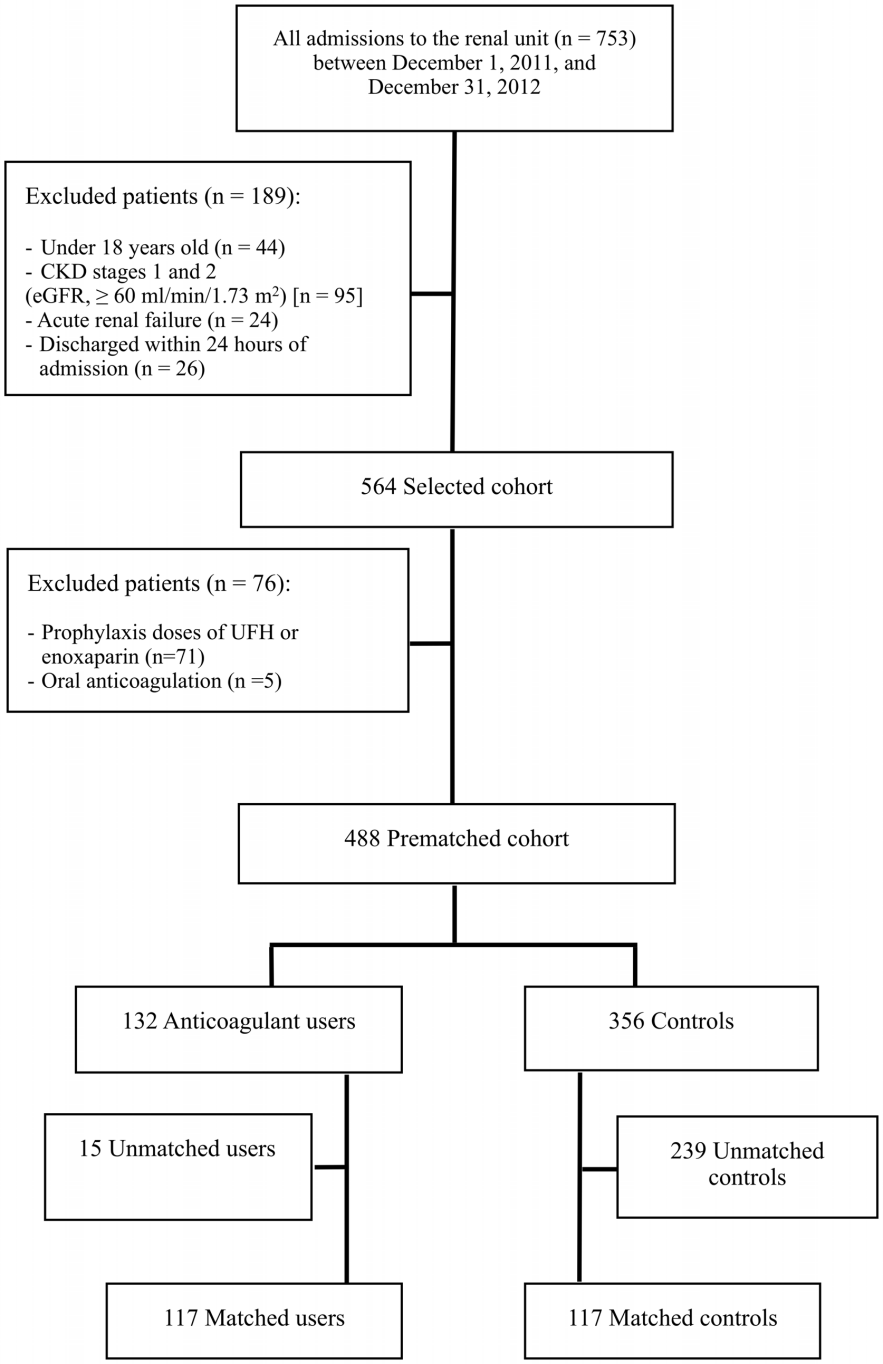
Cohort creation.

During the study period, a total of 488 patients with CKD stages 3 to 5 (estimated glomerular filtration rate [GFR], 10–59 ml/min/1.73 m^2^) fulfilled the inclusion criteria of the study. (Figure 1) Of these, 132 (27%) received anticoagulation therapy during hospital stay. The mean (SD) duration of anticoagulation therapy was 3.5 (0.2) days for UFH, and 4.2 (0.3) days for enoxaparin (P = 0.410).

### Baseline Characteristics

Baseline characteristics for patients with and those without anticoagulant use are reported in Table 1. Patients who received anticoagulants were older with the mean (SD) age of 67 (13) years, versus 58 (16) years in the non anticoagulant use group (P<0.001); were more likely to have a history of vascular disease (58% vs 41%; P = 0.001), and a history of gastrointestinal bleeding (20% vs 12%; P = 0.030). Moreover, patients who received anticoagulants had lower serum level of albumin, with the mean (SD) serum level of albumin of 3.25 (0.70) g/dL, versus 3.60 (0.62) g/dL in the non anticoagulant use group (P<0.001); and had lower serum level of platelet counts, with the mean (SD) serum level of platelet counts of 185.61 (123)×10^3^/µL, versus 225.99 (106) ×10^3^/µL in the non anticoagulant use group (P<0.001).

**Table 1:** Baseline characteristics of patients with and without anticoagulant use.

	No. (%) of Participants		
	Anticoagulant Use		
Characteristics	Treated (n = 132)	Untreated (n = 356)	*P* Value
**Demographics**			
Age, mean (SD), y	67 (13)	58 (16)	<0.001
Female sex	56 (42)	152 (43)	0.957
Male sex	76 (58)	204 (57)	0.957
**Comorbid conditions**			
Diabetes	104 (79)	256 (72)	0.133
Hypertension	128 (97)	322 (90)	0.021
Vascular disease^a^	77 (58)	146 (41)	0.001
Ischemic stroke	18 (14)	44 (12)	0.760
Anaemia	66 (50)	164 (46)	0.475
History of gastrointestinal bleeding	27 (20)	44 (12)	0.030
Liver cirrhosis	8 (6)	28 (8)	0.564
Charlson Comorbidity Index score, mean (SD)	3.87 (1.21)	3.14 (1.21)	<0.001
**Laboratory data**			
GFR, mL/min/1.73 m^2^			
Baseline, mean (SD)	17.83 (14)	12.16 (11)	<0.001
30–59	26 (20)	35 (10)	0.005
15–29	30 (23)	56 (16)	0.082
<15	76 (58)	265 (74)	0.001
Serum creatinine, mean (SD), mg/dL	4.48 (3.29)	7.05 (4.59)	<0.001
Serum albumin, mean (SD), g/dL	3.25 (0.70)	3.60 (0.62)	<0.001
Serum platelet count, mean (SD), 10^3^/µL	185.61 (123)	225.99 (106)	<0.001
**Medication Use**			
Aspirin	62 (47)	136 (38)	0.097
Clopidogrel	51 (39)	80 (22)	0.001
Aspirin and clopidogrel	36 (27)	50 (14)	0.001
NSAID	3 (2)	4 (1)	0.395

Abbreviations: GFR, glomerular filtration rate; NSAID, non-steroidal anti-inflammatory drug; SD, standard deviation.

SI conversions: To convert serum creatinine to µmol/L, multiply by 88.4.

a Vascular disease is defined as presence of coronary artery disease or peripheral vascular disease.

### Propensity-Based Matching

From the initial cohort of 132 patients with anticoagulant use, 117 were selected using propensity score matching. In the propensity score matched analysis, 15 patients remained unmatched and were thus excluded from the analysis. Prematching characteristics widely differed between those with anticoagulant use and those without anticoagulant use but propensity score matching led to an adequate balance for all characteristics considered (Table 2). The absolute standardized differences for all variables were less than 10%, indicating an adequate postmatch balance.

**Table 2:** Characteristics of patients with and without anticoagulant use after propensity matched analysis.

	No. (%) of Participants		
	Anticoagulant Use After Matching		
Characteristics	Treated (n = 117)	Untreated (n = 117)	*P* Value
**Demographics**			
Age, mean (SD), y	66 (14)	66 (14)	0.969
Female sex	52 (44)	50 (43)	0.895
**Comorbid conditions**			
Diabetes	91 (78)	90 (77)	0.876
Hypertension	113 (97)	112 (96)	0.734
Vascular disease^a^	65 (56)	67 (57)	0.895
Ischemic stroke	15 (13)	17 (14)	0.849
Anaemia	60 (51)	53 (45)	0.475
History of gastrointestinal bleeding	23 (20)	16 (14)	0.293
Charlson Comorbidity Index score, mean (SD)	3.79 (1.3)	3.76 (1.3)	0.838
**Laboratory data**			
GFR, mean (SD), mL/min/1.73 m^2^	15.73 (13)	16.55 (13)	0.616
Serum albumin, mean (SD), g/dL	3.30 (0.7)	3.36 (0.7)	0.535
Serum platelet count, mean (SD), 10^3^/µL	188 (85)	188 (85)	0.977
**Medication Use**			
Aspirin and clopidogrel	28 (24)	28 (24)	1.000

Abbreviations: GFR, glomerular filtration rate; SD, standard deviation.

a Vascular disease is defined as presence of coronary artery disease or peripheral vascular disease.

### Outcome Analyses

**Figure 2:**
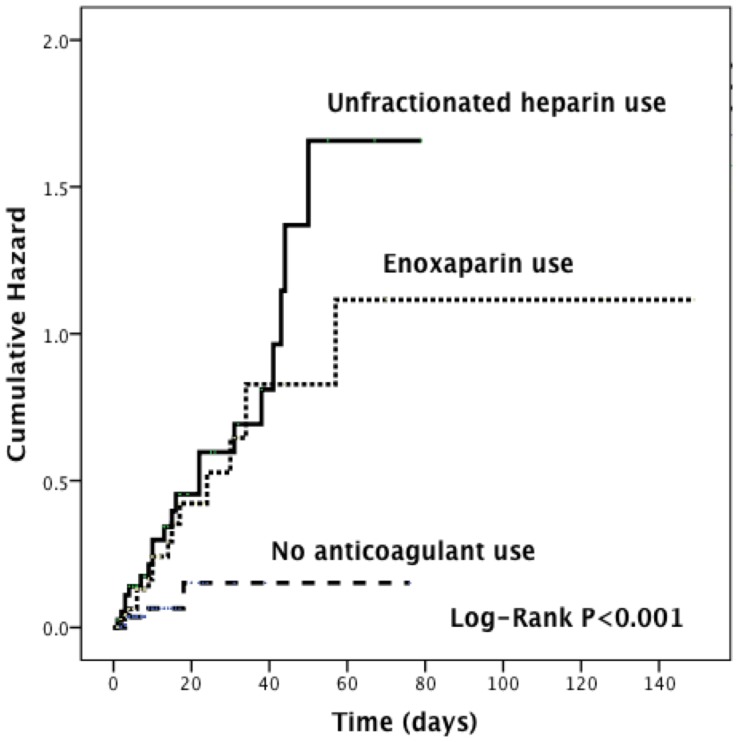
Kaplan-Meier estimates of cumulative hazard of major bleeding events with the use of unfractionated heparin or enoxaparin use.

In this study, 51 major bleeding events were identified. The rate of major bleeding was higher in anticoagulants treated patients than in matched controls (37 vs 5 events, respectively). The hazard ratio for anticoagulants exposure was 4.61 (95% confidence interval [CI], 2.05–10.35) (Table 3). Compared with enoxaparin users, patients who received anticoagulation therapy with UFH had a higher risk of major bleeding (hazard ratio [HR], 4.79 [95% CI, 1.85–12.36]; Figure 2, and Table 4). Furthermore, compared with those who did not receive anticoagulants, patients who did had higher in-hospital mortality (HR, 2.54 [95% CI, 1.03–6.25]; Table 3); longer length of hospital stay (HR, 1.04 [95% CI, 1.01–1.06]; Table 3); and higher hospital readmission at 30 days (HR, 1.79 [95% CI, 1.10–2.91]; Table 3).

**Table 3:** Crude and propensity adjusted hazard ratios of anticoagulant-related adverse outcomes.

	**Major bleeding events**		
	**No. of Events/No. of Patients**		
	**No Anticoagulant**	**Anticoagulant Use**	**HR (95% CI)**
Crude analysis	9/356	42/132	5.48 (2.61–11.51)
Propensity analysis	5/117	37/117	4.61 (2.05–10.35)
	**In-hospital mortality**		
	**No. of Events/No. of Patients**		
	**No Anticoagulant**	**Anticoagulant Use**	**HR (95% CI)**
Crude analysis	8/356	23/132	2.96 (1.27–6.91)
Propensity analysis	3/117	21/117	2.54 (1.03–6.25)
	**Length of hospital stay**		
	**Median (IQR), days**		
	**No Anticoagulant**	**Anticoagulant Use**	**HR (95% CI)**
Crude analysis	4 (6)	8 (15)	1.05 (1.03–1.07)
Propensity analysis	5 (6)	8 (14)	1.04 (1.01–1.06)
	**Readmission at 30 days**		
	**No. of Events/No. of Patients**		
	**No Anticoagulant**	**Anticoagulant Use**	**HR (95% CI)**
Crude analysis	44/356	50/132	1.72 (1.13–2.62)
Propensity analysis	15/117	44/117	1.79 (1.10–2.91)

Abbreviations: CI, confidence interval; HR, hazard ratio; IQR, interquartile range; SD, standard deviation.

**Table 4:** Propensity adjusted hazard ratio of major bleeding events with the use of unfractionated heparin or enoxaparin.

Anticoagulant	No. of Events (% of Patients)	HR (95% CI)	*P* Value
UFH	20 (51)	4.79 (1.85–12.36)	0.001
Enoxaparin	17 (22)	2.10 (1.36–3.24)	0.001

Abbreviations: CI, confidence interval, HR, hazard ratio; UFH, unfractionated heparin.

### Sensitivity Analyses

As presented in Table 5, significant interactions were detected between anticoagulant use and age, sex, presence of diabetes, hypertension, vascular disease, anaemia, estimated GFR, serum level of platelet counts, and use of dual antiplatelet agents. The risk of bleeding associated with the use of anticoagulants was high in individuals older than 65 years (HR, 2.99; 95% CI, 1.70–5.28); in male sex (HR, 1.87; 95% CI, 1.05–3.31); presence of diabetes mellitus (HR, 3.64; 95% CI, 1.80–7.35); hypertension (HR, 3.88; 95% CI, 1.86–8.09); vascular disease (HR, 3.91; 95% CI, 2.18–7.02); anaemia (HR, 2.01; 95% CI, 1.11–3.64); estimated GFR less than or equal to 30 mL/min/1.73 m^2^ (HR, 3.41; 95% CI, 1.62–7.16); serum platelet counts less than or equal to 150×10^3^/µL (HR, 4.52; 95% CI, 2.44–8.39); and use of dual antiplatelet agents (HR, 2.32; 95% CI, 1.21–4.46).

**Table 5:** Hazard ratios of major bleeding events with anticoagulant use stratified by cohort characteristics.

Subgroups	No. of Events/No. Of Patients	HR (95% CI)	*P* value for Interaction
All patients	37/117	4.61 (2.05–10.35)	
sAged ≥65 y	22/64	2.99 (1.70–5.28)	<0.001
Aged 18–65 y	15/53	0.96 (0.49–1.89)	
Male sex	20/65	1.87 (1.05–3.31)	0.028
Female sex	17/52	1.69 (0.92–3.10)	
Diabetes	34/91	3.64 (1.80–7.35)	<0.001
No diabetes	3/26	1.10 (0.39–3.13)	
Hypertension	35/113	3.88 (1.86–8.09)	<0.001
No hypertension	2/4	0.87 (0.20–3.72)	
Vascular disease^a^	27/65	3.91 (2.18–7.02)	<0.001
No vascular disease	10/52	0.66 (0.32–1.34)	
Anaemia	24/60	2.01 (1.11–3.64)	0.018
No anaemia	13/57	1.64 (0.89–3.02)	
GFR ≤30 mL/min/1.73 m^2^	36/102	3.41 (1.62–7.16)	0.001
GFR 30–59 mL/min/1.73 m^2^	1/15	1.96 (0.69–5.59)	
Platelet count ≤150×10^3^/µL	29/52	4.52 (2.44–8.39)	<0.001
Platelet count >150×10^3^/µL	8/65	0.61 (0.29–1.27)	
Dual antiplatelets^b^	9/28	2.32 (1.21–4.46)	0.009
No dual antiplatelets	28/89	1.83 (0.98–3.43)	

Abbreviations: CI, confidence interval; GFR, glomerular filtration rate; HR, hazard ratio; UFH, unfractionated heparin.

a Vascular disease is defined as presence of coronary artery disease or peripheral vascular disease.

b Dual antiplatelets is defined as dual use of aspirin and clopidogrel.

### The Risk Factors for Major Bleeding Events

The frequency of risk factors for bleeding between UFH and enoxaparin was statistically equivalence in all of subgroup evaluated (Table 6). However, after receiving anticoagulants, patients who received UFH had a lower serum level of platelet counts, with the mean (SD) serum level of platelet counts of 139.95 (113)×10^3^/µL in the UFH use group, versus 205.56 (123) ×10^3^/µL in the enoxaparin use group (P<0.001).

**Table 6:** Frequency of risk factors for major bleeding between unfractionated heparin and enoxaparin users.

	No. (%) of Participants		
	Anticoagulant Use		
Variable	UFH (n = 39)	Enoxaparin (n = 78)	*P* Value
Aged ≥65 y	19 (49)	45 (58)	0.432
Male sex	21 (54)	44 (46)	0.845
Diabetes	30 (77)	61 (78)	0.875
Hypertension	37 (95)	76 (97)	0.600
Vascular diseasea	24 (61)	41 (53)	0.431
Anaemia	23 (59)	37 (47)	0.327
GFR ≤30 mL/min/1.73 m^2^	36 (92)	66 (85)	0.380
Platelet count ≤150×103/µL	22 (56)	30 (38)	0.078
Dual antiplateletsb	6 (15)	22 (28)	0.169

Abbreviations: UFH, unfractionated heparin.

a Vascular disease is defined as presence of coronary artery disease or peripheral vascular disease.

b Dual antiplatelets is defined as dual use of aspirin and clopidogrel.

## Discussion

In this prospective observational study of hospitalized patients with moderate to severe CKD, exposure to anticoagulants in recommended doses was associated with a range of adverse outcomes. Major bleeding occurred in 1 in 3 patients who received anticoagulation therapy during their hospital stay. This rate of major bleeding is higher than that of the large trials of anticoagulants [13], [21]–[26]. Noticeably, these large trials excluded CKD patients and renal function of randomized subjects was not reported. Results from this study are consistent with the previous observational study that showed a similar rate of bleeding in patients with severe CKD [11].

In this study, the risk of major bleeding was higher with UFH compared to enoxaparin. Despite the fact that enoxaparin is dependent on the kidney for its elimination and that it can bioaccumulate with reduced kidney function; this did not result into higher bleeding rates according to our findings. The increased rate of bleeding observed with UFH may be attributed to the inhibition of platelet function and increase in vascular permeability; properties that are independent to anticoagulant effects [8]. Unlike UFH, enoxaparin binds less to platelets because of its smaller molecular size and hence has fewer incidences of heparin-induced thrombocytopenia and bleeding events. This is of particular concern because patients with advanced CKD are already more susceptible to bleeding from uraemia-related platelet dysfunction [8].

In a study by Thorevska and colleagues [11], a retrospective medical record review of 620 patients with an estimated GFR of <60 ml/min/1.73 m^2^, which compared the rates of bleeding in patients who received anticoagulation therapy with full-therapeutic dose of UFH, or with enoxaparin, authors reported that the rates of major bleeding increased for both UFH and enoxaparin therapy at each stage of CKD, suggesting that factors other than drug clearance is responsible for anticoagulant bleeding complications. More recently, another retrospective observational study of 7721 dialysis patients who received thrombophylaxis therapy with either UFH or enoxaparin, was able to confirm Thorevska and colleagues [11] results that enoxaparin was not associated with higher bleeding risk in comparison with UFH (risk ratio, 0.98; 95% CI 0.78–1.23), concluding that thrombophylaxis doses of enoxaparin appeared to be safe and could be used as an alternative to UFH in dialysis patients [27]. Of note in the studies mentioned above [11], [27], enoxaparin doses were not reduced to account for kidney function that resulted in bleeding events compared to UFH, whilst in our study, enoxaparin was administrated in adjusted therapeutic doses to CKD patients, who were associated with lower bleeding events compared to UFH. The results of our study highlight the safety of enoxaparin if administered in therapeutic doses with dose adjustment to patients with advanced CKD.

In our study, in-hospital mortality occurred in 1 in 5 patients who received anticoagulation therapy during their hospital stay. This result is comparable with those of Koo and colleagues [28], who in a prospective cohort study investigated the association between anticoagulant usage and mortality in 101 patients admitted with major bleeding during anticoagulation with warfarin, unfractionated heparin or low molecular weight heparin. They reported that at 60 days, the overall mortality was 18%; 6 patients (21%) with excessive warfarin therapy, 5 patients (39%) with UFH or LMWH alone therapy, and 7 patients (60%) UFH or LMWH as a bridge to warfarin therapy. Moreover, the length of hospital stay was longer among anticoagulant users compared with those with no anticoagulation therapy. This fact, in part, can be related to in-hospital bleeding-related complications. At least two-thirds of anticoagulant users had 30 days readmission. This rate of readmission was higher compared with those with no anticoagulation therapy.

Similar to any observational study, this investigation has a number of limitations. Although, through propensity and sensitivity analysis, the effect of observed cofounders were adjusted, there might be a number of unobservable factors that could only be controlled with a randomized controlled trial. In addition, the limited sample size of this study could have resulted in some bias in the results produced. Finally, this study was performed in one hospital, which may also limit the generalizability of the results.

In conclusion, anticoagulation therapy in hospitalized patients with CKD is significantly associated with an increased risk of major bleeding and in-hospital mortality. Higher risk was observed in a range of patient groups and was not reduced after adjusting for the common cofounders. These results suggest that to reduce the risk of bleeding associated with anticoagulation therapy further preventive measures such as laboratory monitoring and/or dose adjustment are warranted.

## References

[1] Coresh J, Selvin E, Stevens LA, Manzi J, Kusek JW, et al (2007) Prevalence of Chronic Kidney Disease in the United States. JAMA 298: 2038–2047. 17986697 10.1001/jama.298.17.2038

[2] Hallan SI, Coresh J, Astor BC, Åsberg A, Powe NR, et al (2006) International comparison of the relationship of chronic kidney disease prevalence and ESRD risk. J Am Soc Nephrol 17: 2275–2284. 16790511 10.1681/ASN.2005121273

[3] Zhang L, Zhang P, Wang F, Zuo L, Zhou Y, et al (2008) Prevalence and factors associated with CKD: a population study from Beijing. Am J Kidney Dis 51: 373–384. 18295053 10.1053/j.ajkd.2007.11.009

[4] Jalal DI, Chonchol M, Targher G (2010) Disorders of hemostasis associated with chronic kidney disease. Semin Thromb Hemost 36: 34–40. 20391294 10.1055/s-0030-1248722

[5] Boccardo P, Remuzzi G, Galbusera M (2004) Platelet dysfunction in renal failure. Semin Thromb Hemost 30: 579–589. 15497100 10.1055/s-2004-835678

[6] Dager WE, Kiser TH (2010) Systemic anticoagulation considerations in chronic kidney disease. Adv Chronic Kidney Dis 17: 420–427. 20727512 10.1053/j.ackd.2010.06.002

[7] Sherman DG, Albers GW, Bladin C, Fieschi C, Gabbai AA, et al (2007) The efficacy and safety of enoxaparin versus unfractionated heparin for the prevention of venous thromboembolism after acute ischaemic stroke (PREVAIL Study): an open-label randomised comparison. The Lancet 369: 1347–1355. 10.1016/S0140-6736(07)60633-3 17448820

[8] Hirsh J, Bauer KA, Donati MB, Gould M, Samama MM, et al (2008) Parenteral AnticoagulantsAmerican College of Chest Physicians Evidence-Based Clinical Practice Guidelines. Chest Journal 133: 141S–159S. 10.1378/chest.08-0689 18574264

[9] Verbeeck R, Musuamba F (2009) Pharmacokinetics and dosage adjustment in patients with renal dysfunction. Eur J Clin Pharmacol 65: 757–773. 19543887 10.1007/s00228-009-0678-8

[10] British Medical Association, Royal Pharmaceutical Society of Great Britain(2012) British National Formulary. London: BMA, RPS, (No 64).

[11] Thorevska N, Amoateng-Adjepong Y, Sabahi R, Schiopescu I, Salloum A, et al (2004) Anticoagulation in hospitalized patients with renal insufficiency: a comparison of bleeding rates with unfractionated heparin vs enoxaparin. Chest Journal 125: 856–863. 10.1378/chest.125.3.856 15006942

[12] Schulman S, Beyth RJ, Kearon C, Levine MN (2008) Hemorrhagic Complications of Anticoagulant and Thrombolytic Treatment. American College of Chest Physicians Evidence-Based Clinical Practice Guidelines. Chest Journal 133: 257S–298S. 10.1378/chest.08-0674 18574268

[13] Cohen M, Demers C, Gurfinkel EP, Turpie AG, Fromell GJ, et al (1997) A comparison of low-molecular-weight heparin with unfractionated heparin for unstable coronary artery disease. Efficacy and Safety of Subcutaneous Enoxaparin in Non-Q-Wave Coronary Events Study Group. N Engl J Med 337: 447–452. 9250846 10.1056/NEJM199708143370702

[14] Prism P (1998) Study Investigators. Inhibition of the platelet glycoprotein IIb/IIIa receptor with tirofiban in unstable angina and non-Q-wave myocardial infarction. N Engl J Med 338: 1488–1497. 9599103 10.1056/NEJM199805213382102

[15] Rubin DB (2001) Using propensity scores to help design observational studies: application to the tobacco litigation. Health Serv Outcomes Res Methodol 2: 169–188.

[16] Thoemmes F (2012) Propensity score matching in SPSS. http://arxivorg/abs/12016385 Accessed March 18, 2013.

[17] Brookhart MA, Schneeweiss S, Rothman KJ, Glynn RJ, Avorn J, et al (2006) Variable selection for propensity score models. Am J Epidemiol 163: 1149–1156. 16624967 10.1093/aje/kwj149 PMC1513192

[18] Pisters R, Lane DA, Nieuwlaat R, de Vos CB, Crijns HJGM, et al (2010) A Novel User-Friendly Score (HAS-BLED) To Assess 1-Year Risk of Major Bleeding in Patients With Atrial Fibrillation The Euro Heart Survey. Chest Journal 138: 1093–1100. 10.1378/chest.10-0134 20299623

[19] Deyo RA, Cherkin DC, Ciol MA (1992) Adapting a clinical comorbidity index for use with ICD-9-CM administrative databases. J Clin Epidemiol 45: 613–619. 1607900 10.1016/0895-4356(92)90133-8

[20] Austin PC, Grootendorst P, Anderson GM (2007) A comparison of the ability of different propensity score models to balance measured variables between treated and untreated subjects: a Monte Carlo study. Stat Med 26: 734–753. 16708349 10.1002/sim.2580

[21] Levine M, Gent M, Hirsh J, Leclerc J, Anderson D, et al (1996) A comparison of low-molecular-weight heparin administered primarily at home with unfractionated heparin administered in the hospital for proximal deep-vein thrombosis. N Engl J Med 334: 677–681. 8594425 10.1056/NEJM199603143341101

[22] Merli G, Spiro TE, Olsson C-G, Abildgaard U, Davidson BL, et al (2001) Subcutaneous enoxaparin once or twice daily compared with intravenous unfractionated heparin for treatment of venous thromboembolic disease. Ann Intern Med 134: 191–202. 11177331 10.7326/0003-4819-134-3-200102060-00009

[23] Blazing MA, de Lemos JA, White HD, Fox KAA, Verheugt FWA, et al (2004) Safety and Efficacy of Enoxaparin vs Unfractionated Heparin in Patients With Non–ST-Segment Elevation Acute Coronary Syndromes Who Receive Tirofiban and Aspirin. JAMA 292: 55–64. 15238591 10.1001/jama.292.1.55

[24] Antman EM, McCabe CH, Gurfinkel EP, Turpie AGG, Bernink PJLM, et al (1999) Enoxaparin Prevents Death and Cardiac Ischemic Events in Unstable Angina/Non–Q-Wave Myocardial Infarction Results of the Thrombolysis In Myocardial Infarction (TIMI) 11B Trial. Circulation 100: 1593–1601. 10517729 10.1161/01.cir.100.15.1593

[25] Decousus H, Leizorovicz A, Parent F, Page Y, Tardy B, et al (1998) A clinical trial of vena caval filters in the prevention of pulmonary embolism in patients with proximal deep-vein thrombosis. N Engl J Med 338: 409–416. 9459643 10.1056/NEJM199802123380701

[26] Antman EM, Morrow DA, McCabe CH, Murphy SA, Ruda M, et al (2006) Enoxaparin versus Unfractionated Heparin with Fibrinolysis for ST-Elevation Myocardial Infarction. N Engl J Med 354: 1477–1488. 16537665 10.1056/NEJMoa060898

[27] Chan KE, Thadhani RI, Maddux FW (2013) No difference in bleeding risk between subcutaneous enoxaparin and heparin for thromboprophylaxis in end-stage renal disease. Kidney Int 84: 555–561. 23677243 10.1038/ki.2013.152

[28] Koo S, Kucher N, Nguyen PL, Fanikos J, Marks PW, et al (2004) The effect of excessive anticoagulation on mortality and morbidity in hospitalized patients with anticoagulant-related major hemorrhage. Archives of internal medicine 164: 1557. 15277289 10.1001/archinte.164.14.1557

